# Early-Life Socialization Enhances Social Competence and Alters Affiliative Preference in Piglets

**DOI:** 10.3390/ani15233395

**Published:** 2025-11-24

**Authors:** Lu Luo, Zhengyu Li, J. Elizabeth Bolhuis, Yuyan Wang, Dongsheng Wu, Yansen Li, Chunmei Li

**Affiliations:** 1 College of Animal Science & Technology, Nanjing Agricultural University, Nanjing 210095, China; 2022010@njau.edu.cn (L.L.); li18536494005@163.com (Z.L.); yuyanwang2023@163.com (Y.W.); 15375038932@163.com (D.W.); liyansen@njau.edu.cn (Y.L.); 2 Adaptation Physiology Group, Department of Animal Sciences, Wageningen University & Research, 6700 AH Wageningen, The Netherlands

**Keywords:** comingling, aggression, regrouping, social test, welfare

## Abstract

Early-life socialization (comingling with other litters before weaning) may affect social behaviors in pigs, but whether the impacts are sex-dependent is unknown. We assessed the effects of early-life socialization on interactions and behavioral responses of female and male pigs in a controlled test setting. Piglets (N = 12 litters) were either comingled from 14 days of age till weaning (Socialized, SOC) or not (Control, CON). Pigs (n= 96) were weaned at 28 days of age and regrouped in mixed-sex groups of eight. At 40 days of age, pigs were individually placed in an arena with one pen mate and one unfamiliar pig of the same sex kept behind fences. Behaviors (exploring the environment, exploring the fences, social contact, aggression, etc.) were observed for 8 min. Generalized linear mixed models with treatment and sex as fixed and pen as random effects were used. Overall, males spent more time exploring the fences of conspecifics than females. Although aggression was displayed by only a small minority of pigs overall, it was more frequently directed towards unfamiliar pigs. Despite this low prevalence, SOC pigs (10.4%) were significantly less likely to show aggression towards strangers than CON pigs (27.7%) (*p* = 0.0217), with this effect observed irrespective of sex. Critically, a time-dependent treatment-by-sex interaction emerged for the time spent exploring the pen mate’s fence. While the interaction was only a trend over the full 8 min (*p* = 0.0889), it became statistically significant during the latter 4 min of the test (*p* = 0.049). This interaction was observed, with SOC-males spending more time on this behavior than SOC-females and all CON pigs, potentially indicating a time-specific and possible shift in social preference. General exploratory and locomotor behaviors (including walking, standing, and general exploration of the environment) were not or only minimally influenced by treatment or sex. In conclusion, early socialization has a significant impact on reducing aggression towards unfamiliar conspecifics. However, the effects on social preference and interactions appear sex-dependent, possibly indicating an enhanced preference for familiar pen mates in males under specific testing conditions.

## Simple Summary

Early-life socialization of piglets—achieved by allowing interaction with neighboring litters before weaning—is an effective and low-cost strategy to improve pig welfare. While instances of aggression were rare overall, our study found that this practice significantly reduced the proportion of pigs exhibiting such behavior towards unfamiliar conspecifics later in life, suggesting it enhances social competence and lowers the threshold for threat perception. Importantly, these effects appeared sex-dependent but nuanced: socialized males exhibited a stronger social preference for their familiar pen-mates over strangers, which was mainly evident in the latter half of the test rather than being a uniform pattern. This suggests that early social experiences play a crucial role in establishing a more robust social support system, particularly for males. Implementing this simple management change can lead to smoother social mixing, fewer stress-related injuries, and better welfare outcomes in commercial pig production.

## 1. Introduction

In commercial pig farms, practices such as abrupt weaning and regrouping disrupt established social structures, often leading to increased aggression, injury, and compromised welfare [1,2]. These challenges can be exacerbated by the limited opportunity for piglets to interact with peers beyond their litter before weaning [3]. Pre-weaning socialization, which allows piglets from different litters to mix before weaning, has been proposed as a strategy to mitigate these adverse effects by promoting the development of social skills during a critical developmental period [2,4].

Early-life socialization mimics natural conditions, where piglets begin interacting with non-littermates around two weeks of age [5,6]. Studies consistently indicate that this early exposure improves pig welfare. Specifically, piglets with socialization experience display reduced post-weaning aggression and adapt more quickly to new social groups [2,5,7]. The mechanism for this improvement remains complex. Some studies attribute the reduced aggression and skin injuries observed during regrouping to enhanced social familiarity, as pigs were mixed with familiar conspecifics [8,9]. However, other studies have also reported decreased aggression and injuries even when pigs were mixed with unfamiliar individuals [2,5,6]. This suggests that socialized pigs may acquire enhanced social competence, enabling them to interact positively with new individuals, including completely unfamiliar ones, through learning. Although pigs clearly differentiate between familiar and unfamiliar conspecifics [10], most existing early socialization research has focused on behaviors during standard regrouping events. Therefore, it remains unclear whether early socialization differentially influences their interaction and behavior toward familiar versus unfamiliar conspecifics in a controlled choice test setting, which is crucial for distinguishing between familiarity effects and enhanced social competence.

Therefore, the current study aimed to investigate how early-life socialization influences pigs’ social interactions and behaviors with familiar versus unfamiliar conspecifics using a test. To this aim, pigs exposed to early-life socialization or not were individually tested. Behaviors and their preference towards a pen-mate or an unfamiliar pig were observed. We hypothesized that early-life socialization would influence behavior in several ways: (1) socialized piglets would demonstrate reduced aggression and increased non-aggressive interactions towards both familiar and unfamiliar pigs; (2) socialization would increase males’ preference for being close to conspecifics; and (3) the effects would be more pronounced in males, particularly resulting in socialized males being less aggressive and having more positive interactions with conspecifics compared with control males, given that males typically show stronger hierarchical and aggressive behaviors [11,12]. Males are also known to engage more actively in social exploration and interactions, making them potentially more sensitive to changes in social opportunities.

## 2. Materials and Methods

### 2.1. Animals and Housing

**Figure 1:**
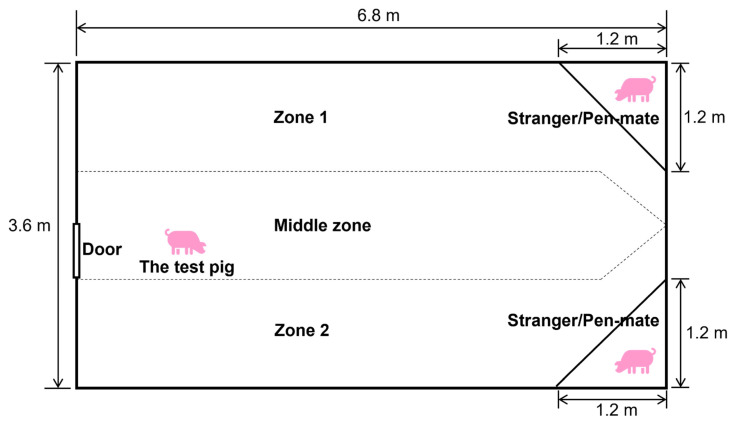
Schematic illustration of the experimental arena used for the Novel Peer Test.

In this experiment, piglets (Duroc × (Landrace × Yorkshire)) from 12 litters were studied. Piglets were either socialized with another litter before weaning (referred to as SOC, below) or not (referred to as CON). Sows (parity: 1st) were inseminated on the same day and housed in a research farm in Taizhou, Jiangsu province, China, from 1 month before farrowing. One week before the expected farrowing date, they were moved to individual farrowing pens. Piglets were cross-fostered within treatment if litter sizes exceeded 14. Litter size at 13 days of age (SOC: 11.3 ± 0.8, CON: 11.0 ± 1.3 piglets/litter) and at weaning (SOC: 11.2 ± 0.9, CON: 10.7 ± 1.2 pigs/litter, n = 131 piglets at weaning), and weaning age (SOC: 27.7 ± 0.5, CON: 27.7 ± 0.2 days) did not differ between treatments. Piglets were not tooth resected, castrated, or tail docked. Farrowing pens (1.8 m × 2.2 m) had a slatted floor with a small solid area (0.3 m × 2.2 m) for heating. Each pen had one drinking nipple for the piglets and one for the sow. Additionally, a rope was provided in each pen for the piglets as enrichment. Sows were kept in farrowing crates. A jute sack was placed at the side-front for each sow and it was changed every week. Sows were fed a standard commercial diet twice a day. The room temperature was set at 25 °C and gradually decreased to 21 °C over a period of 2 weeks. In the first two weeks after birth, one heating lamp was provided in each pen. Each pen was cleaned daily, and lights were on from 7:00 until 19.00 h (Figure 1).

From 14 days of age, the SOC piglets were brought together by opening a small door (24 × 40 cm) located at the back of the two farrowing pens.

Piglets were weaned at 28 days of age. A total of 96 pigs were selected and regrouped into 12 new pens, with eight pigs per pen. All pigs within a new pen had been exposed to the same treatment (Socialized or Control) but originated from different farrowing pens (including pens beyond the immediately neighboring ones for SOC piglets). The composition of each new group was balanced for sex and body weight. Each pen measured 2.05 m × 1.75 m and had a slatted floor. Each pen had one drinking nipple and pigs received solid commercial feed ad libitum. On the weaning day, the temperature was set at 25 °C and was decreased over the course of 2 weeks to 21 °C. In the first week after weaning, one heating lamp was provided for each pen. The lights were on from 7:00 to 19:00.

### 2.2. Social Preference Test

To assess the behaviors and interactions towards a familiar versus an unfamiliar pig (see Table 1), a test was carried out around 40 days of age. One pig from the CON group was not exposed to the test due to health problems. Therefore, a total of 95 pigs were included in this test.

Pigs were individually placed in a 6.8 × 3.6 m test arena with solid walls away from their home pen (Figure 1). All pigs had been in the arena once before for a novel environment test at 37 or 38 days of age. This prior exposure likely reduced generalized novelty-related responses during the current social preference test, allowing us to better isolate social behaviors. The test arena was uniformly illuminated, maintained under quiet conditions, and observers were positioned outside the pig’s line of sight to minimize external disturbance. Before the test pig entered the test arena, a pen-mate of the test pig and an unfamiliar pig (referred to as a stranger) from a pool of 20 pigs in the farm that were not included in the treatment groups were placed in the corners of the arena in a triangular area behind a fence, until both pigs were habituated to the new environment (no escaping, struggling, or high-pitched vocalizations). These holding zones were enclosed by fixed metal fences, forming triangular areas that were approximately 1.2 × 1.2 m in size. The fences were angled to create a clear barrier separating the stimulus pigs from the center of the test arena. Adaptation occurred within 5 min for all pigs, except for one pig, which kept escaping and struggling and was excluded from the test. The sex of the familiar and unfamiliar pigs placed in the corners was the same as that of the tested pigs. Each pig was used as a stranger four or five times. To control for potential habituation or stress-related changes due to repeated use, the identity of the unfamiliar pig was systematically rotated across different test days and treatment groups.

The pen-mate and the stranger were placed in the two opposing corners. The assignment of the pen-mate and the stranger to the left versus right corner was systematically alternated for each test pig (i.e., balanced across all subjects) to avoid the influence of the different locations. After the pen-mate and the strange pig were habituated to the environment, the test pigs could enter the test arena through the door. Once a pig entered the test arena from the door, the door was shut behind it, and a timer was started. Pigs stayed in the test arena for 8 min in total and left the test arena through the door and went back to their home pen. The test was carried out over three days, and the test order was balanced for treatment, pen, and sex.

Behavior of the pigs (Table 1) was scored continuously using behavior sampling with The Observer XT 16 software (Noldus Information Technology b.v., the Netherlands) (See Table 1 for the ethogram). The same observer did the recording.

### 2.3. Statistical Analysis

Urinating (n = 13 pigs in the whole test) and defecating (n = 20 pigs in the whole test) were very rare, and they were summed as “excretory behavior”. To explore the fences and social contact, the time directed at the pen-mate as a proportion of the total time spent on these behaviors was calculated.

SAS (SAS 9.4, SAS Institute Inc., Cary, NC, USA) was used for all statistical analysis.

The time spent on exploring the fences, social contact, the total time spent in exploring the fence and social contact of either conspecifics as a proportion, the total time spent in exploring the fence of conspecifics, the total time spent in social contact with the conspecifics, walking, standing and exploring the environment and frequency of excretory behavior were analyzed with a linear mixed model, with socialization treatment, sex and their interactions as fixed effects, and pen nested within treatment as random effect. Litter was not included as an additional random effect because all treatment groups were established at the pen level after weaning, and preliminary analysis confirmed that including litter did not significantly alter the primary results. The latency of the pigs to explore the fence of the pen-mate and stranger, and social contact with the pen-mate and the stranger were also analyzed using the same model. The latencies were log-transformed, and the excretory behavior was square-root transformed to obtain normality of residuals. Following all transformations, model assumptions, including the normality and homogeneity of residuals, were carefully checked and met.

Lying, other postures, and standing alert were not observed frequently and were therefore analyzed as 0–1 variables using generalized linear mixed models with a binary distribution and a logit link function. The occurrence of aggression directed at the stranger and pen-mate was 0 for one of the treatment × sex combinations; therefore, these variables were analyzed as a 0–1 variable using Fisher’s exact tests to compare treatment groups. All variables were tested for the entire test duration of 8 min, as well as for the first and last 4 min of the test. This split was specifically chosen to detect potential shifts in social behavior or preference that might occur as the test pig habituated to the novel arena or as motivation changed over time.

Significant interactions (*p* < 0.05) and the tendencies in the interactions (0.05 < *p* < 0.10) were further investigated with post hoc pairwise comparisons using the least square means. We interpreted results falling within the 0.05 < *p* < 0.10 range as non-significant trends, and these were discussed cautiously to avoid over-interpretation. Results are presented as mean and SEM.

## 3. Results

General behaviors during the test unrelated to social interactions, i.e., time spent exploring the environment, proportion of pigs standing alert, and frequencies of defecating and urinating were not affected by treatment or sex both during the entire test (see Supplementary Table S1) or during the first or last part of the test. The same held for time spent standing or walking, and proportion of pigs lying or sitting/kneeling (Supplementary Table S1).

### 3.1. The Entire Test (8 Min)

**Figure 2:**
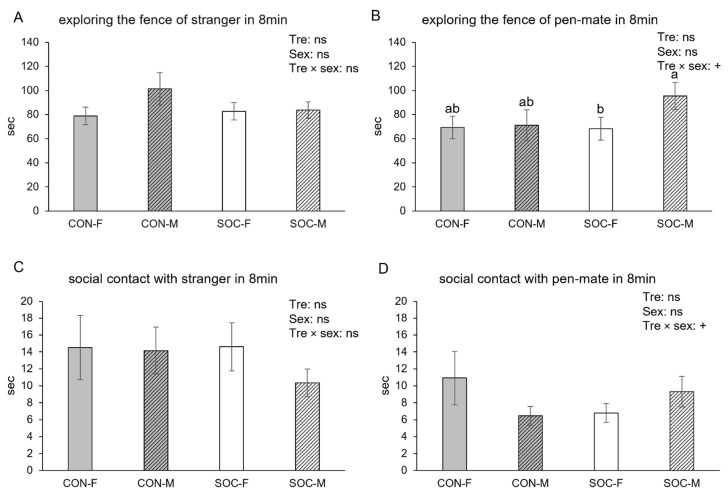
Time spent on exploring the fence of the stranger (**A**) and the pen-mate (**B**), and time spent on social contact with stranger (**C**) and pen-mate (**D**) during the entire test for female (F) and male (M) pigs from the control (CON) and socialized (SOC) groups. Statistical significance of treatment (Tre), sex and their interaction (Tre × sex) is indicated. 0.05 < + *p* < 0.10 and non-significance is ns. Groups lacking a common letter (a, b) significantly differ (*p* < 0.05).

The total time spent exploring the fence near either one of the pigs was affected by sex (female: 149.7 ± 7.3, male: 176.0 ± 8.8 s, *p* = 0.0079). This overall sex difference was consistent across both the first 4 min and the last 4 min of the test, indicating a stable sex difference in general conspecific interest. The duration (Figure 2A) and latency of exploring the fence of the stranger were not affected by treatment or sex. Time spent exploring the fence of the pen-mate showed a non-significant trend for an interaction between treatment and sex (*p* = 0.0889; Figure 2B). This trend was not supported by consistent significant differences in the pairwise comparisons across all groups or time windows (Figure 2B), indicating that the interaction was marginal for the 8 min period. Exploring the fence of the pen-mate as a proportion of the total time spent exploring fences of either pig was not affected by treatment or sex.

The duration or latency of social contact with either one of the pigs, time spent in social contact with the stranger (Figure 2C), or social contact with the pen-mate as a proportion of social contact with either pig was not affected by treatment or sex. However, the absolute time spent in social contact with the pen-mate showed a non-significant trend toward a treatment × sex interaction (*p* = 0.0723). Importantly, although this interaction approached significance statistically, subsequent pairwise comparisons did not reveal any significant differences between groups, suggesting the trend did not translate into meaningful group-level effects.

Aggression did not occur often during the test. Overall, aggressive behavior was very uncommon among all tested pigs. However, more pigs showed aggression towards the unfamiliar pig (n = 20) than towards their pen-mate (n = 3). SOC pigs were less likely to show aggression towards the stranger than CON pigs (SOC: 10.4%, CON: 27.7%, *p* = 0.0217), whereas sex or its interaction with treatment did not affect the aggression (CON-M: 14.9%, CON-F: 12.8%, SOC-M: 4.2%, SOC-F: 6.3%).

### 3.2. The First Half of the Test

**Figure 3:**
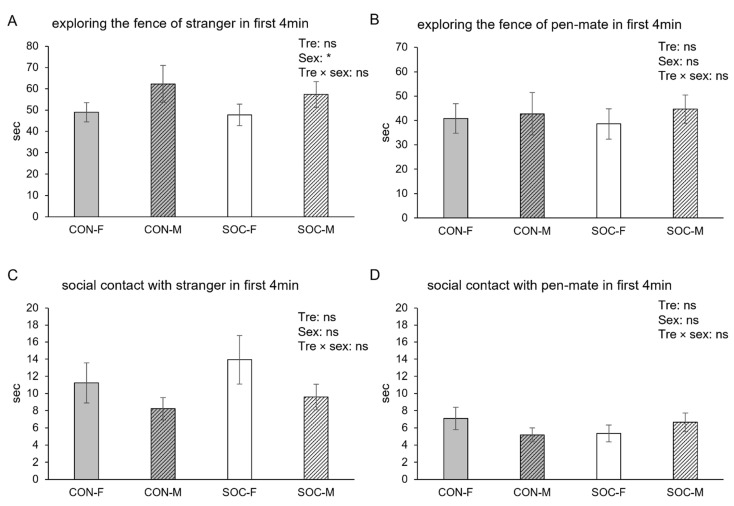
Time spent on exploring the fence of the stranger (**A**) and the pen-mate (**B**), and time spent on social contact with stranger (**C**) and pen-mate (**D**) during the first 4 min of the test for female (F) and male (M) pigs from the control (CON) and socialized (SOC) groups. Statistical significance of treatment (Tre), sex and their interaction (Tre × sex) is indicated. * *p* < 0.05 and non-significance is ns.

In contrast to the full 8 min test period, the first 4 min showed minimal evidence of treatment effects. The total time spent exploring the fence near either one of the pigs tended to be affected by sex (female: 86.8 ± 4.5, male: 97.3 ± 4.5 s, *p* = 0.056). The time spent exploring the fence of the stranger was influenced by sex (female: 48.4 ± 3.4, male: 59.7 ± 5.2 s, *p* = 0.0251, Figure 3A). The percentage of pigs showing aggressive behaviors did not differ between SOC and CON pigs (SOC: 10.4%, CON: 17.0%, *p* > 0.10) and was not affected by sex (F: 18.8%, M: 19.1%, *p* > 0.10). The time spent exploring the fence of the pen-mate (Figure 3B), social contact with the stranger (Figure 3C) and social contact with the pen-mate (Figure 3D) were not influenced by any factor.

### 3.3. The Last Half of the Test

**Figure 4:**
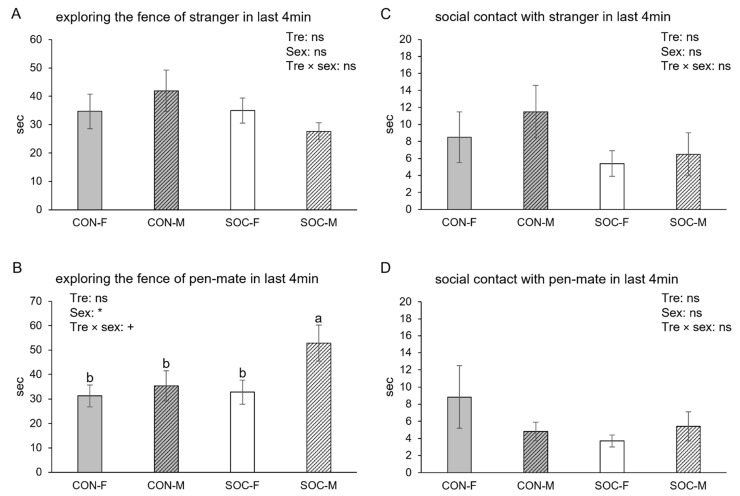
Time spent on exploring the fence of the stranger (**A**) and the pen-mate (**B**), and time spent on social contact with stranger (**C**) and pen-mate (**D**) during the last 4 min of the test for female (F) and male (M) pigs from the control (CON) and socialized (SOC) groups. Statistical significance of treatment (Tre), sex and their interaction (Tre × sex) is indicated. * *p* < 0.05, 0.05 < + *p* < 0.10 and non-significance is ns. Groups lacking a common letter (a, b) significantly differ (*p* < 0.05).

The total time spent exploring the fence near either one of the pigs was not affected by treatment or sex, nor was the time spent exploring the fence of the stranger (Figure 4A); however, the time spent on exploring the fence of the pen-mate was affected by sex (*p* = 0.0472) and the treatment × sex interaction (*p* = 0.049). This interaction effect is time-specific, as it emerged significantly only in the latter half of the test. Pairwise comparisons showed that the SOC-M spent more time exploring the fence of the pen-mate than the other three groups (*p* < 0.05, Figure 4B). Consistent with the entire test, aggression remained infrequent overall in this time segment. However, SOC pigs (4.2%) were less likely to show aggression towards the stranger than CON pigs (17.0%, *p* = 0.0351), with no effect of sex. Time spent on social contact with the stranger (Figure 4C) and with the pen-mate (Figure 4D) was not influenced by any factor.

## 4. Discussion

In this study, we aimed to investigate how early-life socialization, by allowing piglets to comingle with other litters before weaning, influences their behaviors and interactions with both familiar and unfamiliar conspecifics in a test setting. Notably, general behaviors such as time spent exploring the environment, walking, excretory behavior, and standing alert were not affected by treatment or sex. This confirms that the observed differences in social behaviors and preferences cannot be attributed to treatment- or sex-related changes in overall activity levels, general arousal, or stress reactivity, strengthening our conclusion that the social measures reflect true social preference.

Early-life socialization significantly reduced aggression, which was primarily directed toward unfamiliar conspecifics. This finding is consistent with the literature suggesting that diverse pre-weaning social experiences enhance social competence, enabling piglets to navigate novel social contexts with reduced conflict propensity [13]. Previous research, such as work in group lactation systems, has found that social experience leads to shorter fight durations and a reduction in aggression post-weaning [14]. Several mechanisms, supported by existing literature, may explain this improved competence. Furthermore, early socialization may facilitate better coping mechanisms and lower overall stress levels [3], allowing socially experienced pigs to remain calmer when confronted with unfamiliar individuals [15]. Furthermore, this socialization might contribute to them being more adept at interpreting social cues, which mitigates the misunderstandings that often precede aggression [2,16,17].

Aggressive interactions primarily occurred toward unfamiliar pigs, typically when the test pig approached the stimulus animal’s fence. This pattern is likely driven by the fundamental need to establish dominance when unfamiliar pigs are introduced, as the absence of a pre-existing hierarchy necessitates aggressive interactions to determine social ranking [18,19]. In contrast, familiar pen-mates have already established their positions within the hierarchy, reducing the need for further aggression. Critically, we found that the reduction in aggression provided by the socialization intervention was observed irrespective of sex. Contrary to some prior expectations that males exhibit higher aggression, this lack of a sex effect or a Treatment\times\Sex interaction may be attributed to the test setting constraint; The fence separation fundamentally constrained the full expression of aggression, preventing the typical escalation into full-contact fights. Therefore, the measure recorded reflects initiated or attempted aggression rather than the high-intensity agonistic encounters typically observed during free-contact hierarchy formation [20,21]. This methodological limitation, by inhibiting high-intensity escalation, likely contributed to both the extremely low overall aggression rates and the absence of expected sex differences. Given the low overall frequency of aggression (a 0/1 variable), we propose the SOC intervention provides a significant, cross-sex benefit in reducing the initiation of conflict. This effect appears strong enough to override any baseline sex differences in aggressive tendencies specifically within this constrained fence-separation test context.

As expected, a sex effect was found in non-aggressive social exploration. Males spent more time exploring the fence of either pig, suggesting they had higher general motivation to seek social contact compared to females. This aligns with findings that male pigs generally exhibit higher levels of social interactions in early life, including more social play episodes and nudging [22]. Therefore, the greater exploratory time observed in males likely reflects this inherent, sex-specific difference in social motivation. While some literature suggests male pigs are more fearful in novel environments [23], which could potentially intensify their inclination to seek social support, the consistency of our findings with general sex differences in exploratory behavior supports social motivation as the primary driver here.

The most significant finding was that males appeared to be more affected by early-life socialization than females. Specifically, SOC-M spent more time exploring the fence of the pen-mate than the other three groups, particularly in the latter half of the test. This sex-dependent effect is likely rooted in the inherent behavioral sex dimorphism. Males’ heightened social motivation and activity levels [22] may have made SOC-M more receptive to and effective at accumulating social experience during the comingling period, facilitating the formation of stronger social bonds or preferential relationships with their pen-mates [14]. The shift in preference displayed by SOC-M—initially exploring the stranger and then rapidly shifting focus to the pen-mate—may reflect a positive influence of early socialization on their social coping strategy and flexibility. In the challenging test environment, this pattern suggests a possible shift where SOC-M, after exploring the unfamiliar conspecific, may prioritize seeking affiliation with their familiar and trusted pen-mate [24]. This ability to efficiently transition from novelty-seeking (stranger exploration) to affiliation (pen-mate preference) highlights the potential for early socialization to confer greater social competence, which is particularly pronounced in the more socially active male sex.

As this is the first study to investigate these specific behaviors and interactions toward familiar and unfamiliar conspecifics in pigs, certain limitations in the experimental setup should be addressed in future research. Firstly, the design of the test arena could be further optimized. Increasing the distance between the familiar and unfamiliar conspecifics is recommended, as this would reduce proximity-driven influence and give the test pig a more distinct choice between the two social stimuli, thereby allowing for a clearer assessment of its social preference. Secondly, we initially intended to record vocalizations, which provide valuable insights into social needs and emotional states [25,26]. However, accurately determining the source was extremely challenging because calls from the three pigs often overlapped, reverberated, or occurred simultaneously. Future studies should consider using advanced acoustic tracking technology or directional microphones. Thirdly, the influence of the familiar pig’s emotional state posed a challenge. We anecdotally observed that some familiar pigs produced high-pitched vocalizations when the test pig approached the unfamiliar individual, which appeared to lead the test pig to return to them immediately. This suggests that the familiar pig’s social motivation and need for companionship could potentially play a crucial role in shaping the test pig’s choice. Future research should account for these social dynamics by including systematic scoring of stimulus-pig behaviors and evaluating the impact of individual social motivation and even the behavior of the stimulus pigs used in the test.

## 5. Conclusions

This study demonstrates that early-life socialization, achieved through comingling before weaning, reduces aggression towards unfamiliar conspecifics. This effect was observed within a low-baseline context, where overall aggression was rare and constrained by the fence-separation design. Males spent more time near conspecifics than females and their social preference was more influenced by early-life socialization. This sex-dependent effect emerged primarily in the latter four minutes of the test, where socialized males showed a stronger preference for their pen-mates over unfamiliar pigs. In contrast, non-socialized males generally showed a pattern of spending more time exploring strangers. Future research should further explore the underlying neurobiological and behavioral mechanisms driving these effects. Since these mechanisms were not directly measured in the current study, focusing on them in future work will provide the mechanistic underpinning for the observed behavioral changes. Furthermore, experimental designs should be refined to better capture the complexities of social preference in pigs.

## Figures and Tables

**Table 1:** Ethogram used to score the behaviors and location of the pigs during the social preference test.

Behavior	Definition
Posture class	
Standing alert	Standing motionless
Standing	Standing with four paws on the floor
Walking	Walking
Lying	Lying on the floor
Other posture	Sitting or kneeling
Behavior class	
Exploring environment	Exploring the floor or wall of the arena by sniffing, nosing, licking or rooting with the rooting disc, except the areas with the familiar and unfamiliar pigs
Exploring the fence of the pen-mate ^a^	Exploring the fence behind which the pen-mate is placed by sniffing, nosing, licking or rooting with the rooting disc
Exploring the fence of the stranger ^a^	Exploring the fence behind which the unfamiliar pig is placed by sniffing, nosing, licking or rooting with the rooting disc
Social contact with the pen-mate ^a^	Touching or sniffing any part of the pen-mate, including nose contact
Social contact with the stranger ^a^	Touching or sniffing any part of the unfamiliar pig in the fence, including nose contact
Aggression directed at the pen-mate ^a^	Fighting; horizontal or vertical knocking with the head or forward thrusting with the snout towards the pen-mate; intense ramming or pushing the pen-mate; biting the pen-mate
Aggression directed at the stranger ^a^	Fighting; horizontal or vertical knocking with the head or forward thrusting with the snout towards the unfamiliar pig; intense ramming or pushing the unfamiliar pig; biting the unfamiliar pig
Defecating ^b^	Defecation
Urinating ^b^	Urinating
Other behavior	All other behaviors which are not described above

Zone class, posture class and behaviors were scored as states unless indicated otherwise a were also scored as latency; b were scored as events, all other behaviors as states.

## Data Availability

None of the data was deposited in an official repository but was available from the corresponding author upon request.

## Supplementary Materials

The following supporting information can be downloaded at: https://www.mdpi.com/article/10.3390/ani15233395/s1, Table S1 Means ± SEM of the general behaviors during the test unrelated to social interactions or social preferences.
