# Supplementary material for: Early-Life Socialization Enhances Social Competence and Alters Affiliative Preference in Piglets
Source: Animals (Basel). 2025 Nov 24;15(23):3395. doi: 10.3390/ani15233395 (PMC12691441; doi:10.3390/ani15233395)
Supplement: Supplementary file 1 [file animals-15-03395-s001.zip › animals-3984763-supplementary.pdf]

Effects of early-life socialization on social preference and behaviour towards familiar and unfamiliar conspecifics in pigs

L. Luo, Z.Y. Li, J.E. Bolhuis, Y.Y. Wang, D.S. Wu, Y. Y. Li, C.M. Li

animal journal

Supplementary material

Table S1 Means ± SEM of the general behaviours during the test unrelated to social interactions or social preferences

Behaviours	CON-F	CON-M	SOC-F	SOC-M	T	S	T × S
Standing alert (% of pigs)	7.4 of pigs	6.3	11.6	9.5	ns	ns	ns
Standing (sec)	119.5 ± 112.3	107.7 ± 11.0	101.3 ± 7.9	105.8 ± 9.8	ns	ns	ns
Walking (sec)	354.6 ± 17.6	366.6 ± 10.8	370.8 ± 8.8	373.7 ± 10.5	ns	ns	ns
Lying (% of pigs)	3.2	6.3	5.3	2.1			
Other posture (% of pigs)	4.2	6.3	11.6	7.4	ns	ns	ns
Exploring environment (sec)	202.3 ± 16.4	189.9 ± 14.4	205.2 ± 12.6	202.8 ± 16.6	ns	ns	ns
Excretory behaviour (frequency)	1.4 ± 0.4	1.3 ± 0.2	1.3 ± 0.3	1.6 ± 0.4	ns	ns	ns

T represents treatment and S represents sex, and ns means non-significance
